# Induction of apoptosis by in vitro and in vivo plant extracts derived from *Menyanthes trifoliata* L. in human cancer cells

**DOI:** 10.1007/s10616-018-0274-9

**Published:** 2019-01-04

**Authors:** Tomasz Kowalczyk, Przemysław Sitarek, Ewa Skała, Monika Toma, Marzena Wielanek, Dariusz Pytel, Joanna Wieczfińska, Janusz Szemraj, Tomasz Śliwiński

**Affiliations:** 1 Department of Genetics, Plant Molecular Biology and Biotechnology, Faculty of Biology and Environmental Protection, University of Lodz, Banacha 12/16, 90-237 Lodz, Poland; 2 Department of Biology and Pharmaceutical Botany, Medical University of Lodz, Muszynskiego 1, 90-151 Lodz, Poland; 3 Laboratory of Medical Genetics, Faculty of Biology and Environmental Protection, University of Lodz, Pomorska 141/143, 90-236 Lodz, Poland; 4 Department of Plant Physiology and Biochemistry, Faculty of Biology and Environmental Protection, University of Lodz, Banacha 12/16, 90-237 Lodz, Poland; 5 Department of Biochemistry and Molecular Biology, Medical University of South Carolina, Hollings Cancer Center, HCC-709, 86 Jonathan Lucas Street, Charleston, SC 29425 USA; 6 Department of Immunopathology, Chair of Allergology, Immunology and Dermatology, Faculty of Biomedical Sciences and Postgraduate Training, Medical University of Lodz, 90-752, Żeligowskiego 7/9, Lodz, Poland; 7 Department of Medical Biochemistry, Medical University of Lodz, Mazowiecka 6/8, 92-215 Lodz, Poland

**Keywords:** Glioma cells, *Menyanthes trifoliata*, Apoptosis, Genes expression, Flow cytometry, HPLC analysis

## Abstract

*Menyanthes trifoliata* L. has been used in traditional medicine for centuries. It exists in Asia, Europe, North America and in Morocco and is exploited as a remedy for anemia and lack of appetite. This plant shows many pharmacological properties, but its most interesting one is its anti-cancer potential. The present study examines the induction of apoptosis in grade IV glioma cells after treatment with the extracts from aerial part and root of *M. trifoliata* plants derived from in vitro (MtAPV and MtRV, respectively) and from soil (MtAPS and MtRS, respectively) and presents the first comparison of the biological effects of four different extracts of *M. trifoliata* against glioblastoma cells. The root extracts of *M. trifoliata* plants were found to exhibit cytotoxic effects against grade IV glioma cells, but not normal human astrocytes. HPLC analysis demonstrated the presence of various polyphenolic compounds, including sinapinic acid, ferulic acid, syringic acid and vanilic acid. Higher amount of pentacyclic triterpene (betulinic acid) was also found in MtRV extract. The growth inhibition of human grade IV glioma cells mediated by MtRV extract appears to be associated with apoptosis and G2/M phase cell cycle arrest, and altered expression of the pro- and anti-apoptotic genes (*Bax*, *Bcl*-*2*, *Cas*-*3* and *TP53*) and proteins (Bax, Bcl-2, Cas-3 and p53), as well as decreased mitochondrial membrane potential. Our results indicate that *M. trifoliata* gives promising results as an anti-cancer agent for human glioblastoma cell lines. However, further research is necessary in view of its therapeutic use.

## Introduction

Despite the development of very precise diagnostic and therapeutic methods, gliomas remain one of the most challenging diseases of the central nervous system. It has high rates of recurrence and is one of the leading causes of death worldwide (Davis 2016). In Europe alone, 27,000 new cases of malignant astrocytic tumors are diagnosed every year (http://www.rarecarenet.eu/rarecarenet/). However, existing therapies, such as surgical resection followed by adjuvant external beam radiation and chemotherapy, are being constantly improved. Currently, the most commonly-used chemotherapeutics for glioma are temozolomide (Wang et al. 2017), carmustine and PCV (procarbazine, vincristine, lomustine), but these also demonstrate significant toxicity towards healthy tissues. An alternative approach to traditional therapeutic methods is application of anti-cancer compounds derived from plants: there are many chemicals with antineoplastic properties in the plant kingdom and the list of plant species known to have anticancer properties is constantly growing. One of the most interesting groups of plant chemical compounds exhibiting anticancer properties are the phenolic acids including chlorogenic, ellagic, sinapinic, syringic and caffeic (Guimarães et al. 2007; Zhang et al. 2015a, b; Ahire et al. 2017; Deka et al. 2017; Mady and Shaker 2017; Sadeghi Ekbatan et al. 2018). Another biologically-active group of compounds with anticancer properties are the terpenoids. This is a large group of natural compounds derived from C30 precursors including triterpenes, steroids, limonoids, quassinoids, triterpenoidal and steroidal saponins (Sandjo and Kuete 2013). These compounds have considerable potential in the fight against different types of human cancer (Huang et al. 2012), with naturally-occuring terpenoids playing a role in the chemoprevention and therapy of cancer cells. Triterpenes appear to play an important role in this regard: actein, astragaloside IV, cucurbitacin I, ursolic acid and betulinic acid all exhibit cytotoxic properties against various types of tumors (Escandell et al. 2008; Einbond et al. 2009; Qi et al. 2010; Tiwari et al. 2014; Kim et al. 2015). The latter deserves special attention due to its wide antineoplastic effect. Betulinic acid (3b-hydroxy-lup-20(29)-en-28-oic acid) is naturally-occuring pentacyclic lupane-type triterpenoid present in a range of plant species including *Betula alba*, *Eucalyptus* sp., *Lavandula angustifolia*, *Platanus acerifolia*, *Rosmarinus officinalis* (Tiwari et al. 2014) and *Menyanthes trifoliata* (Stabursvik 1953; Janeczko et al. 1990; Patočka 2003). *M. trifoliata* L. (*Menyanthaceae*) is found throughout the northern hemisphere. It is a marsh plant that grows about thirty centimeters high, and offers a range of pharmacological effects including anti-inflammatory or immunomodulating properties (Tunón et al. 1994; Huang et al. 1995; Kuduk-Jaworska et al. 2004) and is a rich source of betulinic acid and triterpene saponines (Patočka 2003).

This study is the first one to investigate the cytotoxic effect, the expression of apoptotic-related genes, and the induction of apoptosis in a human glioma cell line after treatment with extracts prepared from aerial parts and roots derived from in vitro (MtAPV and MtRV) and from soil-grown (MtAPS and MtRS) *M. trifoliata* plants. The main polyphenolic compounds in the tested extracts were identified and quantified by HPLC analysis.

## Materials and methods

### Establishment of in vitro and soil-grown *M. trifoliata* plants

Axenic in vitro culture was set up using *M. trifoliata* seeds. The seeds were surface sterilized as follows: The seeds were placed in 70% ethanol (EtOH) for one minute. After this time, the EtOH was replaced with 30% commercial bleach ACE (Procter&Gamble) and the tubes inverted from time to time for ten minutes. The bleach was then removed and the seeds washed five times for five minutes with sterile water.

**Fig. 1:**
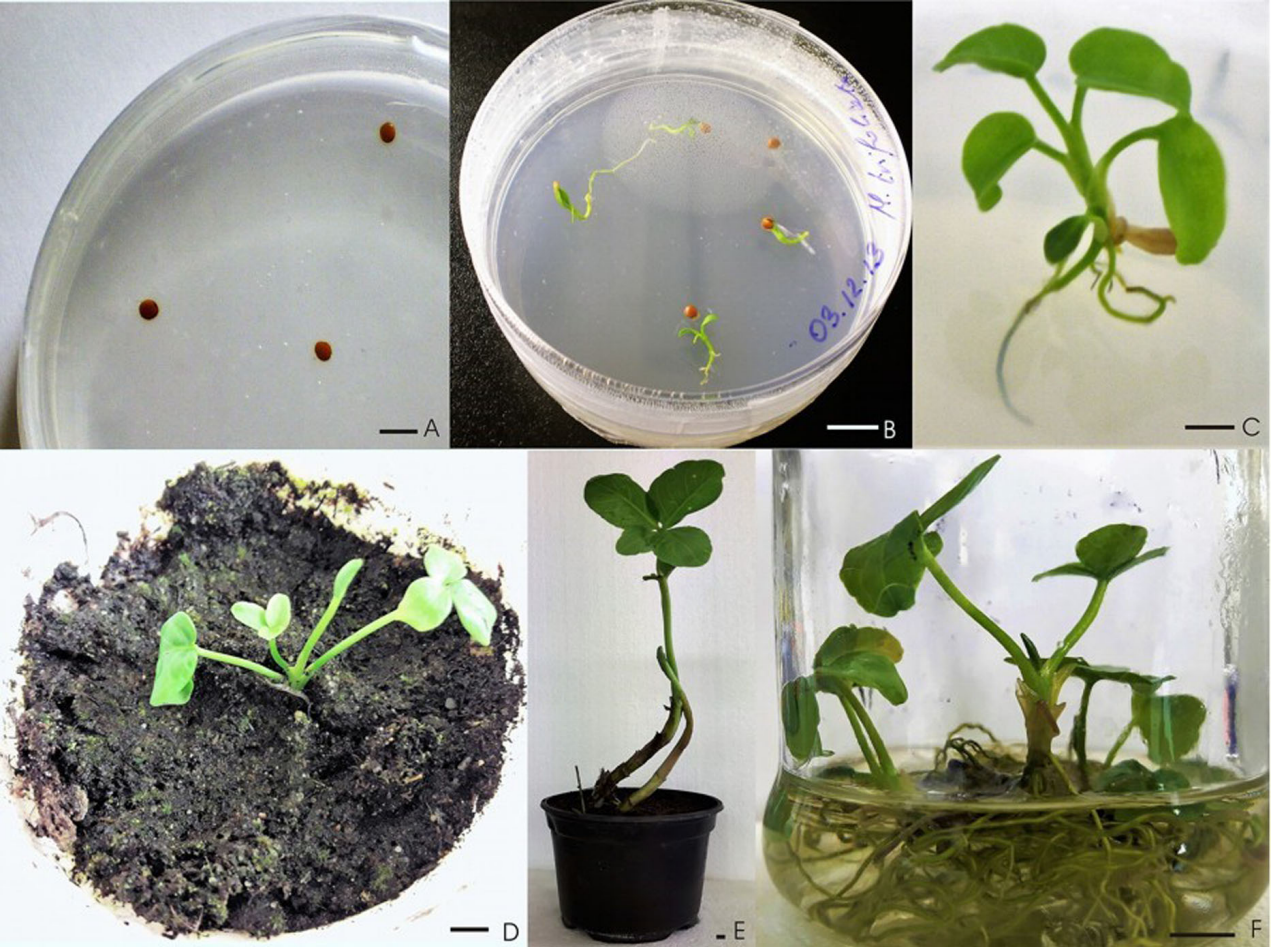
Development stages of *Menyanthes trifoliata* in vitro and in vivo (soil-grown) conditions. **a** Seeds after surface sterilization. **b** Seeds germination after 1 month, **c** 6 weeks old in vitro plant, **d** 3 month old plant in soil, **e***M. trifoliata* after 1 year, **f** plants in vitro cultured in liquid SH medium (bar = 1 cm)

The sterilized seeds were germinated under aseptic conditions on SH medium (Schenk and Hildebrandt 1972) supplemented with vitamins, 50 mg L^−1^ gibberellic acid (GA_3_, Duchefa Biochemie, Haalen, The Netherlands) and 0.02 mg L^−1^ kinetin as described previously (Adamczyk-Rogozinska and Wysokińska 1998) (Fig. 1a). The medium was solidified with 0.8% agar. A sterile hood was used for preparing the culture.

After 10 weeks, the seedlings were transferred to liquid SH medium for culture under the following conditions: 16/8 h light/dark photoperiod, light intensity 40 µmol m^−2^ s^−1^, temperature 26 °C.

The resulting shoot tips were excised and placed on SH medium (0.8% agar, 0.5 mg L^−1^ indole-3-acetic acid (IAA, Duchefa Biochemie), 1 mg L^−1^ 6-benzylaminopurine (BAP, Duchefa Biochemie). After 30 days, the shoot tips were subcultured on solid SH medium with 0.5 mg L^−1^ BAP for shoot elongation. Following this, 1–2 cm shoots were placed on SH with 0.8% agar and 0.5 mg L^−1^ IAA (Duchefa Biochemie, Haalen, The Netherlands) for rooting for 6 weeks. The rooted shoots (Fig. 1c) were then moved to liquid SH medium for further growth. The in vitro plants were subcultured every 4 weeks on new liquid SH medium (Fig. 1f). Plant material in vitro propagation was followed by apical meristem.

The procedure for creating soil-grown plants was as follows: the seeds were planted in a sterile mixture of soil, peat and sand (3:1:1 v/v) (Adamczyk-Rogozinska and Wysokińska 1998) for germination in the greenhouse under the following conditions: temperature 26 °C, 16/8 h light/dark photoperiod, light intensity of 40 µmol m^−2^ s^−1^.

A voucher specimen was deposited at the Department of Genetics, Plant Molecular Biology and Biotechnology, University of Lodz, Poland.

### Plant extract preparation

**Fig. 2:**
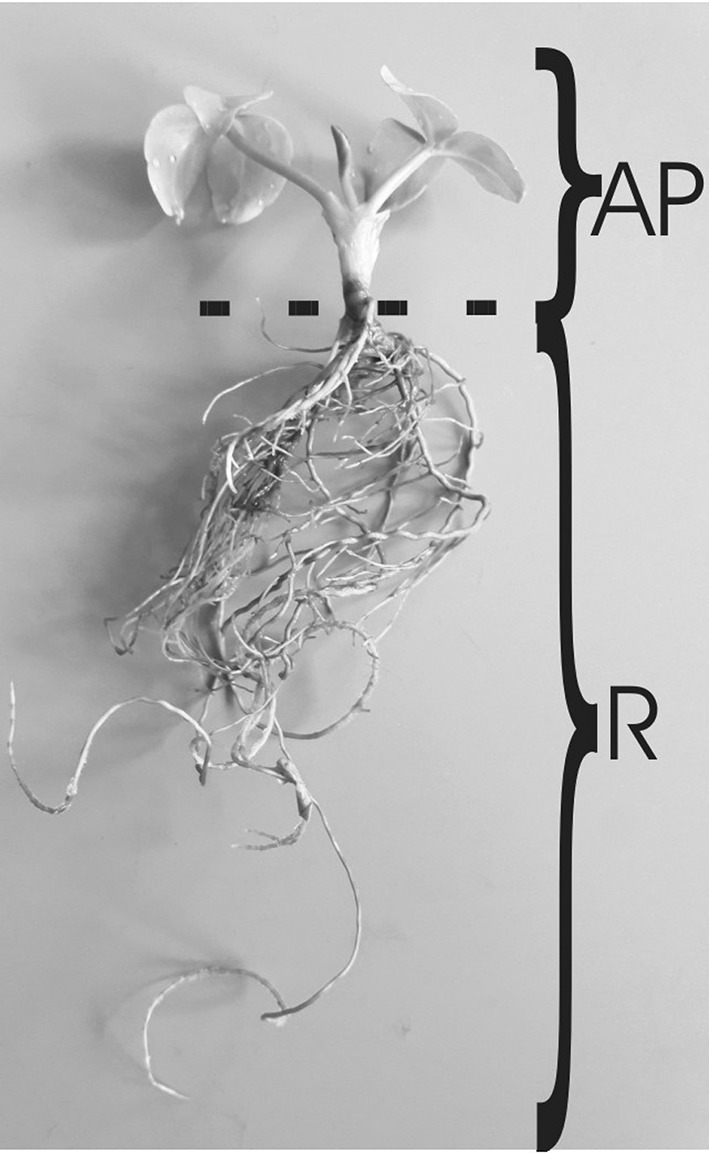
Aerial parts and roots of in vitro *M. trifoliata* (AP aerial parts, R roots)

Four different *M. trifoliata* extracts were used: two from 1-year-old in vitro derived plants (Fig. 1e, f) obtained from the aerial parts (MtAPV) and roots (MtRV) (Fig. 2), and two from plants obtained from soil grown for 1 year in the greenhouse from the aerial parts (MtAPS) and roots (MtRS). Briefly, the extracts were prepared as described previously (Sitarek et al. 2016b). The yields (w/w) of the extracts with regard to initial dry weight of plant material were 52.8% and 50.4% for MtAPV and MtRV for in vitro plants, respectively, and 54.2% and 48.4% for MtAPS and MtRS for soil-grown plants, respectively.

### Phytochemical analysis

The phenolic compounds were examined by HPLC method (Dionex, Sunnyvale, CA, USA) (Sitarek et al. 2016a). The results are presented in Table 1. Identification and quantification of betulinic acid were conducted according to the modified method of Pai et al. (2011). Chromatographic analysis was carried out using HPLC system (Dionex) equipped with a photodiode-array detector. Chromatographic separation was achieved on a Supelcosil RP column (C18, 4.0 mm × 150 mm, 5 µm). joined with a guard column (GOLD Drop-In guards, 10 × 4 mm, 5 µm, Polygen, Gliwice, Poland) at 25 °C. Mobile phase consisting of 16% water (A) and 84% acetonitrile (B) in an isocratic mode with injection volume 20 µl. The flow rate was 1 cm^3^ min^−1^ and the absorbance was measured at 210 nm, the analysis time was 12 min for both standards and plant extracts. Betulinic acid in plant extracts were identified by comparing the retention time and UV spectra of the analyzed samples with the respective data obtained from the analysis of reference standard. Quantification was based on the calibration curve for betulinic acid standard constructed over the range of 5–250 µg cm^−3^, the linearity of the calibration curve was verified by the correlation coefficient (r^2^ = 0.9982).

**Table 1:** The contents of secondary metabolites in *Menyanthes trifoliata* plants obtained in vitro and in vivo cultures

No.	Compounds	MtRV	MtAPV	MtRS	MtAPS
		µg g^−1^ dry weight			
1	Betulinic acid	**5437.15 ± 141.33** ^**c**^	**395.31 ± 14.5** ^**a**^	**3938.95 ± 82.65** ^**b**^	**390.00 ± 15.87** ^**a**^
2	Vanillic acid	2.83 ± 0.16^**c**^	2.23 ± 0.09^**b**^	1.98 ± 0.09^b^	1.43 ± 0.06^a^
3	Syringic acid	**113.80 ± 0.80** ^**c**^	n.d.	**29.96 ± 0.05** ^**b**^	0.98 ± 0.02^a^
4	Ferulic acid	13.80 ± 0.80^**b**^	n.d.	5.09 ± 0.14^a^	n.d.
5	Sinapinic acid	**146.53 ± 7.03** ^**d**^	71.16 ± 3.34^b^	**86.12 ± 5.81** ^**c**^	45.84 ± 1.60^a^
6	*o*-coumaric acid	3.42 ± 0.11^c^	2.95 ± 0.13^c^	2.07 ± 0.09^b^	1.18 ± 0.03^a^
7	Salicylic acid	8.68 ± 0.47^d^	4.21 ± 0.26^b^	5.10 ± 0.27^c^	2.71 ± 0.12^a^
8	Ellagic acid	**518.11 ± 26.46** ^**c**^	**450.65 ± 14.22** ^**b**^	**326.98 ± 20.92** ^**a**^	**298.64 ± 12.54** ^**a**^
9	Coumarin	17.69 ± 0.51^d^	11.37 ± 0.62^c^	5.45 ± 0.30^a^	7.01 ± 37.2^b^
10	t-3-hydroxycinnaminic acid	4.77 ± 0.25^d^	2.03 ± 0.08^c^	0.27 ± 0.008^a^	1.43 ± 0.05^b^
11	Hesperidin	60.73 ± 3.27^c^	70.50 ± 3.80^d^	39.17 ± 3.40^a^	47.59 ± 2.28^b^
12	t-cinnaminic acid	3.98 ± 0.21^c^	3.11 ± 0.12^b^	2.87 ± 0.07^b^	1.89 ± 0.06^a^
13	Hesperetin	43.04 ± 1.54^c^	20.19 ± 0.78^b^	20.94 ± 0.85^b^	11.96 ± 0.56^a^
14	Neochlorogenic acid	11.62 ± 0.66^c^	11.87 ± 0.52^c^	5.70 ± 0.19^a^	7.73 ± 0.40^b^
15	Gentisinic acid	11.65 ± 0.33^b^	35.72 ± 1.28^d^	7.64 ± 0.34^a^	25.46 ± 1.19^c^
16	Chlorogenic acid	**177.34 ± 9.57** ^**b**^	**257.9 ± 14.70** ^**c**^	**128.77 ± 7.21** ^**a**^	**178.13 ± 7.83** ^**b**^
17	Caffeic acid	1.16 ± 0.03^c^	1.31 ± 0.02^c^	0.65 ± 0.28^a^	0.84 ± 0.02^b^
18	1,3-dicaffeoylquinic acid	0.89 ± 0.02^d^	0.22 ± 0.0072^b^	0.35 ± 0.0042^c^	0.15 ± 0.0075^a^
19	*p*-Coumaric acid	10.74 ± 0.44^b^	15.78 ± 0.42^c^	6.89 ± 0.15^a^	9.66 ± 0.41^b^
20	Luteolin	9.08 ± 0.28^d^	7.16 ± 0.27^c^	5.74 ± 0.29^b^	1.68 ± 0.06^a^
21	Rutin	**256.20 ± 3.24** ^**d**^	**152.99 ± 6.42** ^**b**^	**180.04 ± 2.86** ^**c**^	**82.34 ± 3.04** ^**a**^
Sum of phenolic compounds		**1416,06**	**1121,35**	**861,78**	**726,65**

The compounds were determined in 80% aqueous methanol extracts from roots and aerial parts of plants obtained from in vitro (MtRV and MtAPV) and in vivo (MtRS and MtAPS) cultures. Different superscript letter within the rows indicate significant differences in the mean values at *p* < 0.05. The compounds present in the highest concentrations are given in bold

### Cell culture

In this study two cell lines were used. Normal human astrocytes (NHA) (Lonza, Basel, Swizerland, CC-2565) were grown in AGM (Lonza, Basel, Swizerland, CC-3187) medium supplemented according to the manufacturer’s protocol, while grade IV glioma cells were derived from a tumor patient. Briefly, cells were cultured with DMEM medium (ThermoFisher Scientific, CA, USA, Gibco, 31331-028) supplemented with 10% FBS (EuroClone, Pero, MI, Italy) 100 Units/mL penicillin and 100 μg/mL streptomycin under a humidified atmosphere with 5% CO_2_ and 95% air at 37 °C. In all experiments, 4 × 10^5^ cells were seeded per 75 cm^2^ flask. The confirmation of carcinogenic nature of these cells and further procedures are given in a previous study (Sitarek et al. 2016a).

### Cytotoxicity of extracts from in vitro and soil-grown *M. trifoliata* plants

Cytotoxicity of plant extracts was evaluated by MTT assay [3-(4,5-dimethylthiazol-2-yl)-2,5-diphenyltetrazolium bromide] (Thermo Fisher Scientific), which assesses cell metabolic activity according to the manufacturer’s instructions. Cells (1 × 104 cells) were plated in 96-well plates in 100 μL culture medium containing various concentrations (range 0–4 mg mL^−1^) of plant extracts (MtRV, MtRS, MtAPV or MtAPS). 20 μL of MTT solution (5 mg mL^−1^ MTT in PBS) was then added to each well and the plate was incubated in 37 °C until formazan crystals were visible. Then, medium was discarded from wells and the crystals were dissolved in 100 μL of DMSO (Sigma-Aldrich, St. Louis, MO, USA). After 15 min incubation at room temperature plates were read in a microplate spectrophotometer (OMEGA) at 550 nm.

### RNA isolation and quantitative RT-PCR

Briefly, the cells were plated into 6-well culture dishes (3 × 10^5^ cells/well) for 24 h prior to the addition of MtRV plant extract (1.5 mg mL^−1^). The total RNA isolation kit (A&A Biotechnology, Gdynia, Poland) was used to isolate total RNA from cells treated with the plant extracts. The obtained RNA was transcribed into cDNA using TranScriba Kit (A&A Biotechnology). Following this, the expression of four genes (*Bax*, *Bcl*-*2*, *Cas*-*3*, *TP53*) was measured by qRT-PCR using TaqMan^®^ Real-Time PCR Master Mix (Life Technologies, Carlsbad, CA, USA) and Agilent Technologies Stratagene Mx300SP (Santa Clara, CA, USA) working on MxPro software. TaqMan probes (Life Technologies, CA, USA) were used to analyse genes and *18S RNA* (Life Technologies) was included as a reference gene. The PCR was performed as follows: 95 °C for 10 min, 30 cycles of 95 °C for 15 s and 60 °C for 60 s.

### Western blot analysis

Following treatment with MtRV extract for 24 h, glioma cells were harvested, washed with ice-cold PBS (Thermo Fisher Scientific) and lysed in RIPA lysis buffer (Sigma-Aldrich, St. Louis, MO, USA) containing a protease-inhibitor cocktail tablet for 30 min. The supernatant was collected after centrifuging at 18,000 × *g* for 15 min. Total protein was extracted and protein concentration was determined using a bicinchoninic acid assay kit (Thermo Fisher Scientific, CA, USA). For immunoblotting, 30 µg protein from each sample was subjected to 4–20% ExpressPluS PAGE Gel (GenScript, Piscataway, New Jersey, USA) and separated proteins were transferred onto a PVDF membrane using eBlot Protein Transfer (GenScript, Piscataway, NJ, USA). The membrane was blocked with 5% skimmed milk at room temperature for 1 h and then incubated with the primary antibodies against caspase-3, Bcl-2, Bax, p53 (Abcam, Cambridge, MA, USA) and GAPDH (SantaCruz Biotechnology, Santa Cruz, CA, USA), respectively, at 4 °C overnight. After washing, the membrane was incubated with anti-rabbit or anti-mouse HRP-conjugated secondary antibody. GAPDH was used as an internal control to monitor equal protein loading and transfer of proteins from the gel to the membranes. Signals were detected using an enhanced ECL reagent, and BioRad Universal Hood II with Chemiluminescence System (BioRad, Hercules, California, USA). The results shown are representative of three independent experiments.

### Apoptosis/necrosis and cell cycle detection by flow cytometry

In this study, apoptotic and necrotic cells content in grade IV glioma cells was detected using an annexin V-fluorescein isothiocyanate (FITC)/propidium iodide (PI) detection kit (ThermoFisher Scientific, CA, USA) according to the manufacturer’s instructions. Briefly, cells were plated into 6-well culture dishes (2 × 10^5^ cells/well) for 24 h prior to the addition of MtRV extract from *M. trifoliata* plants (1.5 mg mL^−1^). Following 24-h incubation with the tested MtRV extract, the percentage of apoptotic/necrotic cells was determined by the annexin V-FITC/PI assay. The content of glioma cells in different cell cycle phases was accessed using PI/RNase staining buffer (BD Biosciences, San Jose, CA). Cells were seeded as mentioned above and treated of MtRV extract. After 24 h incubation, medium was discarded and cells were collected and fixed with 70% cold ethanol for at least 1 h in − 20 °C. In the next step cells were suspended in staining buffer. Cell analysis was performed using CytoFlex Flow Cytometer (Beckman Coulter).

### Assessment of mitochondrial membrane potential (ΔΨm)

Briefly, the grade IV glioma cells at a concentration of 1 × 10^5^ cells/mL were incubated in 6-well plates for 24 h at 37 °C with 5% CO_2_ with MtRV extract at a concentration of 1.5 mg mL^−1^. JC-1 (5,5′,6,6′-tetrachloro-1,1′,3,3′-tetraethylbenzimidazolyl carbocyanine iodide, Sigma-Aldrich, St. Louis, MO, USA) was used to measure the change in mitochondrial membrane potential (ΔΨm). After trypsinization, the cells were incubated with JC-1 at 37 °C and 5% CO_2_ for 30 min. JC-1 accumulation in the mitochondria is potential dependent and is indicated by a fluorescence emission shift from green to red, with mitochondrial depolarization indicated by a lower ratio of red to green. The resulting fluorescence was measured on a Fluoroskan Ascent plate reader (Thermo Fisher Scientific, CA, USA). Filter pairs of 530 nm/590 nm and 485 nm/538 nm were used (Cossarizza et al. 1993).

### Statistical analysis

Data is presented as mean ± standard deviation (SD). The normality of data was verified by the Shapiro–Wilk test. The Kruskal–Wallis test was used to identify differences between data. Differences with *p* values less than 0.05 were regarded as statistically significant. STATISTICA 13.1 software (StatSoft, Krakow, Poland) was used for all calculations.

## Results

### Identification and quantification of compounds in aerial parts (MtAPV) and roots (MtRV) extracts from in vitro plants and in the aerial parts (MtAPS) and roots (MtRS) extracts from in vivo (soil-grown) plants of *M. trifoliata*

**Fig. 3:**
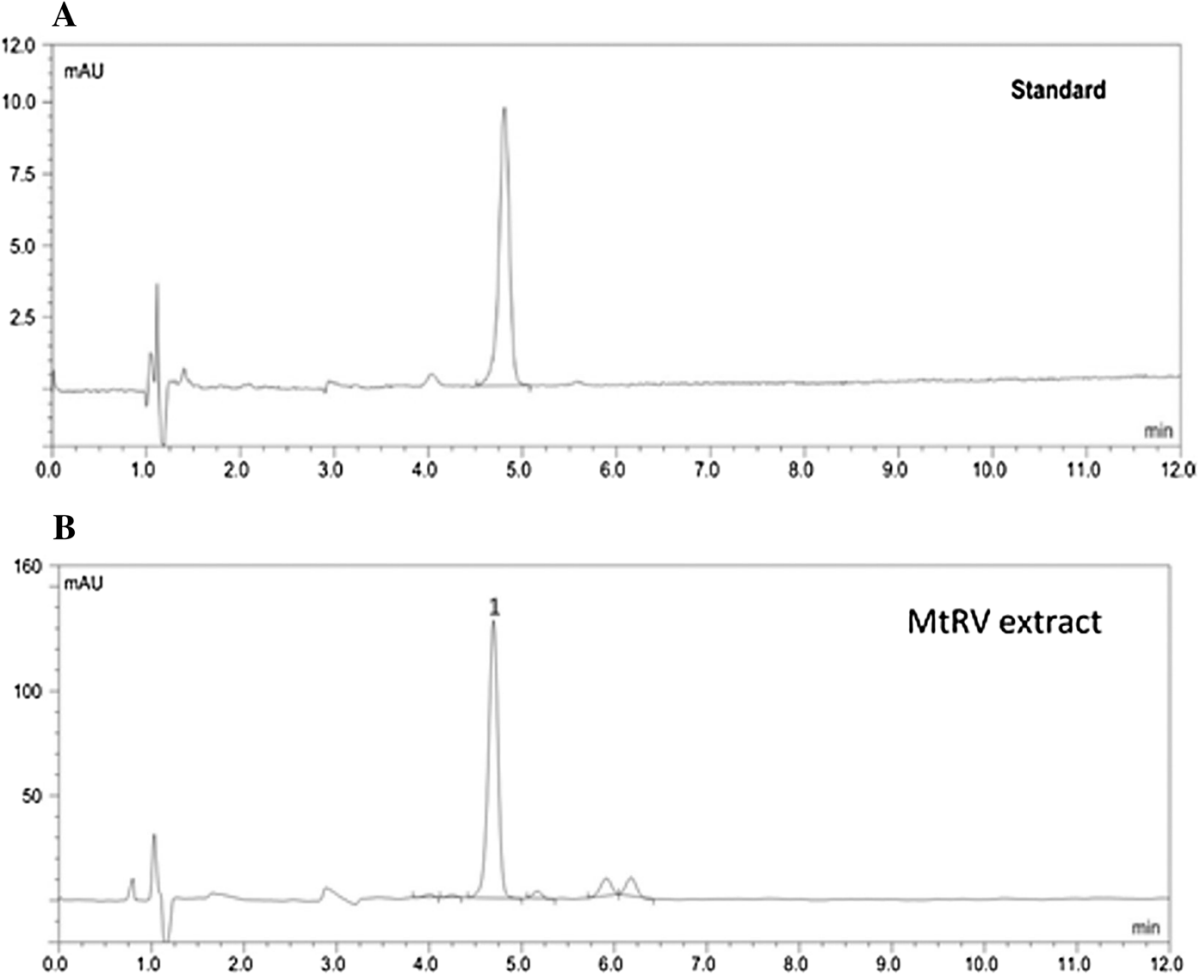
RP-HPLC profile of betulinic acid standard (**a**) and plant extract (**b**), 1 = betulinic acid

**Fig. 4:**
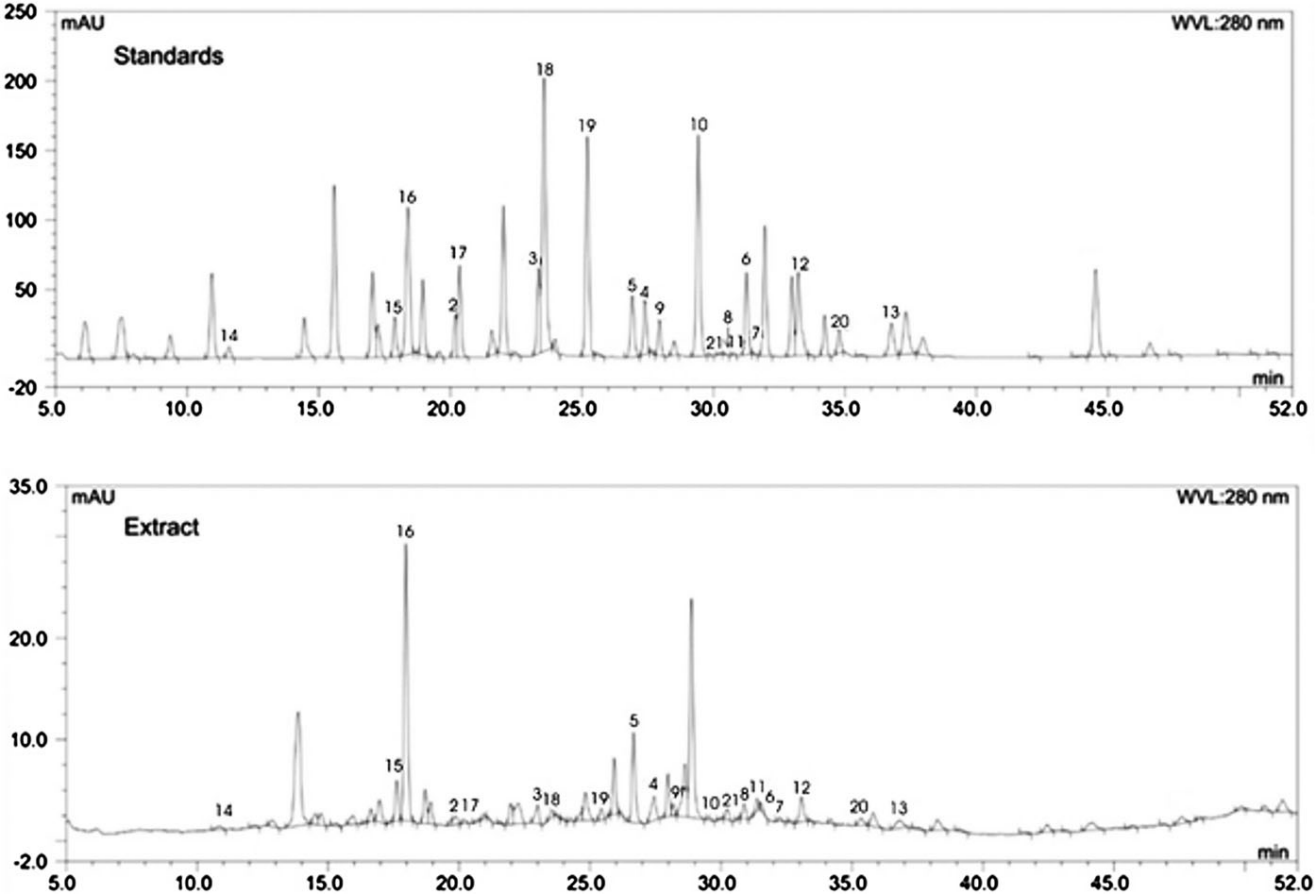
Typical HPLC chromatogram of *Menyanthes trifoliata* in vitro root extract. Numbers correspond to the compound numbers in Table 1

The polyphenolic compounds and pentacyclic triterpenes present in plant extracts of *M. trifoliata* were identified by comparison of retention times, and UV absorption spectra with those of the of authentic standard compounds. In the aqueous methanolic MtAPV, MtRV, MtAPS and MtRS extracts were identified fifteen phenolic acids (chlorogenic acid, synapinic acid, ellagic acid, syringic acid, vanillic acid, *p*-coumaric acid, *o*-coumaric acid, ferulic acid, salicylic acid, t-3-hydroxycinnaminic acid, t-cinnaminic acid, neochlorogenic acid, gentisinic acid, caffeic acid, 1,3-dicaffeoylquinic acid), five flavonoids (rutin, luteolin, hesperetin, hesperidin and coumarin), and one triterpene pentacyclic (betulinic acid) (Table 1). Betulinic acid was accumulated in higher amounts in the root extracts (5437.15 µg g^−1^ DW in MtRV and 3938.96 µg g^−1^ DW in MtRS) than in the extracts from the aerial parts (395.31 µg g^−1^ DW in MtAPV and 390.00 µg g^−1^ DW in MtAPS). The results indicate that the aerial parts of the in vitro and in vivo (soil-grown) plants contained significantly different levels of phenolic acids and flavonoids than the roots (Table 1). In the MtAPV and MtAPS extracts, the polyphenolic fraction was 1121.35 µg g^−1^ DW and 726.65 µg g^−1^ DW, respectively, with ellagic acid, chlorogenic acid and rutin as the main constituents. In turn, in MtRV and MtRS, the polyphenolic fraction was 1416.06 and 861.78 µg g^−1^ DW, respectively, with sinapinic acid, syringic acid and rutin as the major compounds (Table 1). Additionally, other minor compounds were detected in plant extracts: vanillic acid, *p*-coumaric acid, *o*-coumaric acid, ferulic acid, salicylic acid, t-3-hydroxycinnaminic acid, t-cinnaminic acid, neochlorogenic acid, gentisinic acid, caffeic acid, 1,3-dicaffeoylquinic acid, luteolin, hesperetin, hesperidin and coumarin. A typical chromatogram of the aqueous methanolic extract of the *M. trifoliata* plants (MtRV extract) is shown in Figs. 3 and 4. Due to the highest content of polyphenolic compounds and pentacyclic triterpenes contained in the MtRV extract, this extract was chosen for further biological studies.

### Cytotoxic effect of MtRV, MtAPV, MtRS and MtAPS extracts on human grade IV glioma cells and normal human astrocytes

**Fig. 5:**
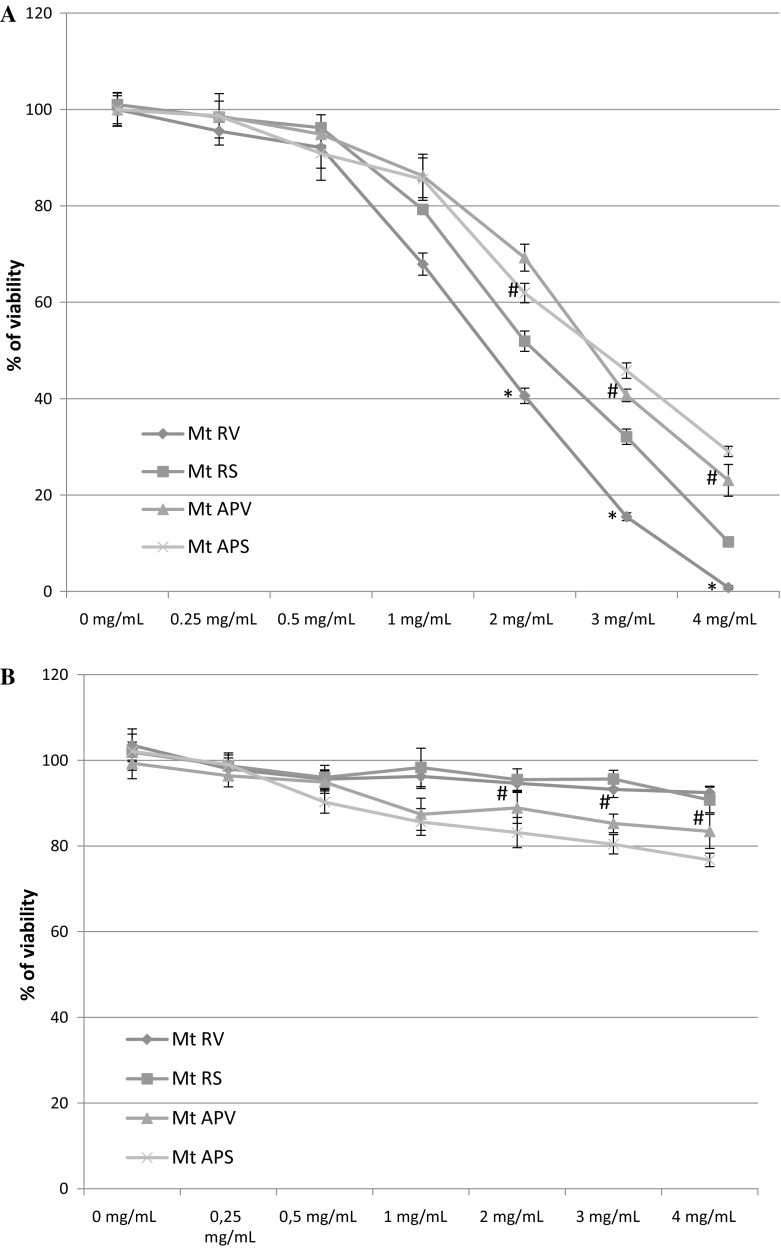
**a** Cell viability of grade IV human glioma cells after treatment with MtAPV and MtRV of in vitro plant extracts and MtAPS and MtRS soil plant extracts of *Menyanthes trifoliata* in various concentrations (0–4 mg mL^−1^). **b** Cell viability of normal human astrocyte (NHA) cells after 24-h treatment with MtAPV and MtRV of in vitro plant extracts and MtAPS and MtRS soil plant extracts of *Menyanthes trifoliata* at various concentrations (0–4 mg mL^−1^) The mean values ± SD were calculated from three independent experiments. MtAPS—aerial parts from soil, MtRS—roots from soil, MtRV—roots in vitro, MtAPV—aerial parts in vitro*. p* < 0.05, #MtAPV versus MtAPS, *MtRV versus MtRS

All tested extracts showed cytotoxic effects in a dose-dependent manner. A higher cytotoxic effect was observed for the MtRV and MtRS (2 mg mL^−1^) extracts than MtAPV and MtAPS (3 mg mL^−1^) but the MtRV extract had a much more potent effect on grade IV glioma cells with IC_50_ 1.5 mg mL^−1^ (Fig. 5a). This extract was chosen for further biological analysis. In addition, no cytotoxic effect was observed on normal human astrocytes (NHA) for any tested extract within the test range (0–4 mg mL^−1^) (Fig. 5b). The survival of these cells after treatment with all tested extracts ranged from 80 to 90%.

### Flow cytometry and cell cycle analysis on grade IV glioma cells co-treated with different concentrations of MtRV extract

**Fig. 6:**
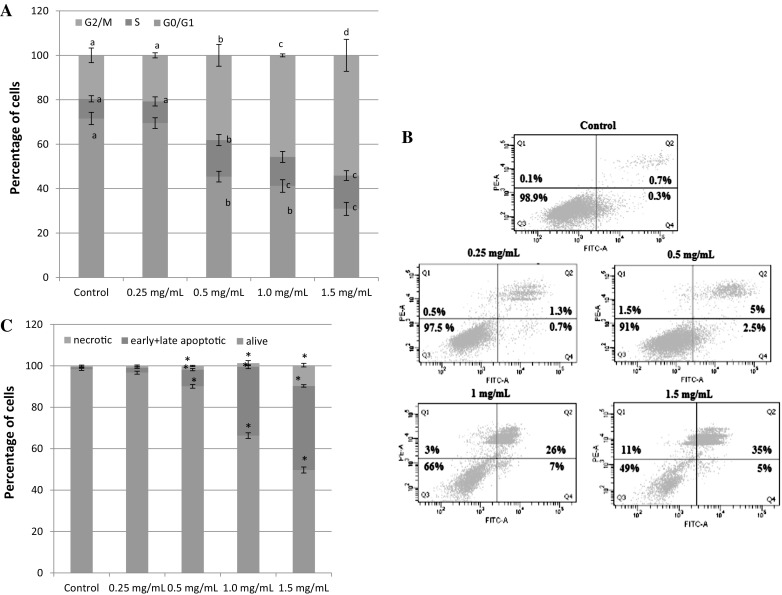
**a** Cell cycle in human glioma cells on IV grade after treatment with MtRV extract of *Menyanthes trifoliata* at various concentrations (0.25–1.5 mg mL^−1^), different letter indicate significant differences in the mean values at *p* < 0.05, **b** representative histograms of apoptosis induction in human grade IV glioma cells after treatment with in vitro root derived plant extract of *Menyanthes trifoliata* at various concentrations (0.25–1.5 mg mL^−1^), **c** diagram present percentage of early and late apoptosis, necrosis and alive cells after 24 h treatment with in vitro root derived plant extract of *Menyanthes trifoliata* at various concentrations (0.25–1.5 mg mL^−1^). The mean values ± SD were calculated from three experiments. *p < 0.05 Mt RV extract versus control

For flow cytometry analysis, the human grade IV glioma cells were treated for 24 h with different concentrations of MtRV extract (0.25, 0.5, 1, 1.5 mg mL^−1^) or DMSO (0.1–0.3%), for controls, and then stained with Annexin-V-FITC and PI. Our results showed an increase in the G2/M phase (*p* < 0.05) associated with the MtRV (0.25–1.5 mg mL^−1^) extract during the 24 h treatment period. A significantly more human grade IV glioma cells in the G2/M phase after treatment with 1.5 mg mL^−1^ MtRV extract was observed (Fig. 6a). The data indicates that inhibition of grade IV glioma cell growth mediated by MtRV extract is associated with G2/M phase cell cycle arrest. Representative results for the human grade IV glioma cells grade are shown in Fig. 6b. The percentage of apoptotic cells increased from 1% in control cells to 40% after treatment with MtRV. This effect showed that the percentage of apoptotic cells was dose-dependent. The most effective concentration (1.5 mg mL^−1^) was chosen to our further studies.

### Expression of apoptosis-related genes in human grade IV glioma cells and loss of mitochondrial membrane potential after treatment with MtRV extract

**Fig. 7:**
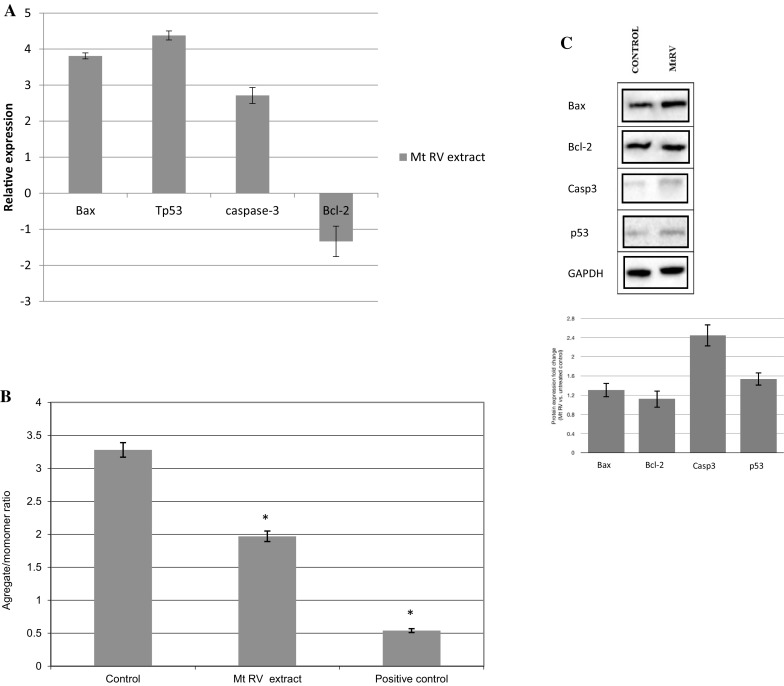
**a** Expression of genes *Bax, Bcl*-*2, Cas*-*3* and *TP53* in human grade IV glioma cells after treatment with 1.5 mg mL^−1^ Mt RV in vitro plant extract of *Menyathes trifoliata*. The transcript level of each gene was normalized to the expression of a reference gene (*18S RNA*). Data is presented as fold change in human grade IV glioma cells after treatment of *Menyanthes trifoliata* extract versus untreated human grade IV glioma cells, in which expression levels of the genes were set as 1. **b** Mitochondrial membrane potential in human grade IV glioma cells after treatment with 1.5 mg mL^−1^ Mt RV in vitro plant extract of *Menyathes trifoliata*. MMP is expressed as 530 nm/590 nm to 485 nm/538 nm (aggregates to monomer) fluorescence ratio, as quantified with a fluorescent plate reader after JC-1 staining). The mean values ± SD were calculated from three independent experiments. *p* < 0.05 control versus MtRV and positive control. **c** Western blot analysis of MtRV extract on Bax, Bcl-2, Casp-3, p53 expression level

To investigate the mechanism of apoptosis induction during 24-h incubation of human grade IV glioma cells with MtRV extract, qRT-PCR was used to evaluate the expression of several apoptosis genes, including *Bcl*-*2, Bax, Cas*-*3 and TP53*. Treatment of the human grade IV glioma cells with 1.5 mg mL^−1^ MtRV extract significantly decreased the mRNA level of *Bcl-2* (*p* < 0.05) (Fig. 7a). In addition, the mRNA level of *Bax, Cas*-*3 and TP53* was significantly increased after 24 h (*p* < 0.05). The levels of apoptosis-related proteins were evaluated by Western blot analysis (Fig. 7c) and changes in their expression were confirmed (Bax, Cas-3 and p53). Additionally, after 24 h, the MtRV extract was found to significantly reduce the level of ΔΨm in human grade IV glioma cells by about 1.5-fold in comparison to the control (Fig. 7b). This result confirms that MtRV extract induces apoptosis through the disruption of mitochondrial membrane potential. This reduction in mitochondrial membrane potential may well initiate the apoptotic cascade in the human grade IV glioma cells treated with MtRV extract of *M. trifoliata* plants.

## Discussion

The plant kingdom is home to many valuable natural products, especially secondary metabolites, that perform a variety of functions in the plant, with some being involved in defence against biotic and abiotic stress or pathogens (Ahmed et al. 2017). The list of known secondary metabolites is extensive: Many exhibit pro-health properties and some of these are used to treat pathologies in humans (Skała et al. 2016). It has been demonstrated that the aqueous extract of *M. trifoliata* is able to induce a suppressive phenotype of dendritic cells that has a reduced capacity to induce Th1 and Th17 stimulation of allogeneic CD4(+) T cells (Jonsdottir et al. 2011). Other data indicate that polysaccharide-rich fractions from *M. trifoliata* play an important role as strong stimulators of immune cells, or as a potent suppressive and anti-inflammatory agents (Kuduk-Jaworska et al. 2004).

Various groups of chemical compounds in *M. trifoliatat* plants have been identified so far (Günter and Krebs 1957; Battersby et al. 1968; Mel’chakova and Kharitonova 1976; Junior 1989; Adamczyk-Rogozinska and Wysokińska 1998). Our findings indicate that total amount of phenolic compounds in the MtRV extract was 1416.06 µg g^−1^ dry weight, while the content in the MtAPV and MtAPS extracts was 1121.35 µg g^−1^ DW and 726.65 µg g^−1^ DW, respectively, and 861.78 µg g^−1^ was present in MtRS. This indicates that *M. trifoliata* is a rich source of many valuable preventive and therapeutic compounds. Betulinic acid is present in the highest concentration in MtRV extract (5437.1 µg g^−1^ DW), which is 1.7-times higher than the content in roots originating from MtRS (3122.1 µg g^−1^ DW) and MtAPV and MtAPS (395.31 µg g^−1^ DW and 390.00 µg g^−1^ DW, respectively). For this reason, root extract from in vitro plants was used for further research in this work. As betulinic acid is known to exhibit anti-tumor properties against various types of cancer cells (Chintharlapalli et al. 2007; Fulda 2008; Reiner et al. 2013), the strong anticancer activity of MtRV extract may be due to its high content of betulinic acid. An important novel aspect of this study is that it is the first paper to compare the contents of biologically-active polyphenolic compounds in different parts of plants derived from in vitro and in vivo (soil-grown) cultures. There is very little data available on the cultivation and regeneration of *M. trifoliata* in vitro, and the study describes the establishment of *M. trifoliata* in vitro cultures and vegetative plant material propagation by apical meristem, which can represent an alternative to organogenesis. A previous study by Adamczyk-Rogozińska and Wysokińska (1998) found that the composition of the culture media, subculture number and the type and concentration of the cytokinin used have a great influence on the shoot multiplication rate of *M. trifoliata* during organogenesis from callus tissue. In our study, application of rooting medium and culture conditions gave very good results, allowing for intensive growth, rooting and development of in vitro plants for further analysis.

The MTT test demonstrated that *M. trifoliata* extracts had a cytotoxic effect against grade IV glioma cells. The best results were observed for MtRV extract (IC_50_ = 1.5 mg mL^−1^) which had the strongest influence on grade IV glioma cells. Additionally, our findings found that none of the tested extracts had a cytotoxic effect against normal human astrocytes (NHA) within the test range (0–4 mg mL^−1^). The survival for all extracts was between 80 and 90%. The MtRV extract was able to inhibit cell growth, arresting cell cycle inthe G2/M phase and finally inducing apoptosis in human glioma cells. The chosen MtRV extract induced apoptosis in a dose-dependent manner and the greatest cytotoxic effect was achieved at a concentration of 1.5 mg mL^−1^. A body of evidence acquired from other studies indicates that the plant extract, its fraction or isolated individual compounds induces G2/M phase arrest and apoptosis in a wide range of cancer cells (Yang et al. 2010; Yan et al. 2011; Kong et al. 2014). Many studies have examined the antitumor activity of betulinic acid (BA) (Fulda 2008; Ali-Seyed et al. 2016). One characteristic property of BA is its ability to induce the mitochondrial pathway of apoptosis in various cancer cells. Betulinic acid also exhibits the ability to regulate the expression of Bcl-2 family proteins (Kumar et al. 2018). This process, known as programmed cell death, plays a crucial role in the development and maintenance of homeostasis in organisms (Henson and Hume 2006). The intrinsic (mitochondrial) pathway of apoptosis is based on the activity of the Bcl-2 family of proteins, and depends on the interaction between proapoptotic and antiapoptotic molecules; this converges on Bax/Bak activation, which controls mitochondrial outer membrane permeability (MOMP).

Our results indicate that treatment of cancer cells with plant extracts results in changes in the expression pattern of apoptosis-related genes. Incubation of the cancer cells in the presence of 1.5 mg mL^−1^ MtRV extract for 24 h simultaneously increased the expression of the *Bax* proapoptotic gene and decreased that of the antiapoptotic *Bcl*-*2* gene. In addition, the *TP53* and *Casp*-*3* genes were found to be up-regulated in glioma cells after exposure to this extract; these proteins play an important role in tumor suppression, cell cycle arrest and the execution-phase of cell apoptosis (Delbridge et al. 2012; Li et al. 2012; Guo et al. 2014). Furthermore, our results also reveal a reduction in mitochondrial membrane potential, which may confirm the presence of an apoptotic cascade in grade IV glioma cells treated with MtRV extract. Based on our findings, indicating changes in the expression of apoptosis-related genes, the observed loss of mitochondrial membrane potential and apoptosis can be attributed to the influence of the higher concentrations of BA present in the MtRV extract, as noted by other authors (Xu et al. 2014; Shankar et al. 2017). Fulda et al. (1998) describe betulinic acid as a cytotoxic agent which induces apoptosis by a directly reducing the transmembrane potential of mitochondria. Mitochondria undergoing permeability transition induced by BA mediate the cleavage of caspase-3 and caspase-8. It was also demonstrated that soluble factors such as cytochrome c or apoptosis-inducing factor released from BA-treated mitochondria are sufficient to initiate the cleavage of caspases and nuclear fragmentation. Previous studies also indicate that BA-treated cancer cells (glioma, melanoma, neuroblastoma) demonstrated upregulation of the pro-apoptotic Bax protein (Fulda et al. 1997; Wick et al. 1999; Selzer et al. 2000), indicating that plant-derived products rich in BA could be potent agents in the fight against cancer cells.

It is interesting to note that the highest content of BA was detected in roots derived from in vitro plants, indicating that this model merits further studies. Additionally, we predict that other compounds contained in the MtRV extract, such as syringic acid, sinapinic acid, ellagic acid, chlorogenic acid and rutin, may play an auxiliary role in the fight against cancer cells acting synergistically. Karthik et al. (2014) evaluated the anticancer activity of syringic acid (SA) in lung carcinoma A549 cell line. They confirmed that there was a significant increase in the percentage of apoptotic cells in the population of SA treated cells. Abaza et al. (2013) found SA to have antimitogenic and chemo-sensitizing activities against human colorectal cancer, thus altering the cell cycle (S/G2-M or G1/G2-M phases) and inducing apoptosis. Our analysis found SA to also be present in the roots of in vitro cultured *M. trifoliata* plants (113.80 µg g^−1^ DW).

Zhao et al. (2013) examined the influence of ellagic acid (EA) on the inhibition of pancreatic cancer growth in Balb C nude mice. It was demonstrated that treatment of PANC-1 xenografted mice with EA resulted in significant inhibition in tumor growth. This phenomenon was associated with suppression of cell proliferation, caspase-3 activation and induction of PARP cleavage. Ellagic acid proved to be effective in inhibition of Bcl-2, cyclin D1, CDK2 and CDK6. This compound also induced the expression of Bax in tumor cells compared to untreated control ones. In another work, the same authors present EA as inducing cytotoxic effects and inhibiting the growth of human bladder cancer cells as a result of cell cycle arrest at the G0/G1 phase, accompanied by apoptotic cell death. Additionally, EA was found to cause the induction of apoptosis through caspase-, ROS- and intrinsic pathways and Bax expression (Ho et al. 2014). EA is also known to demonstrate broad anti-cancer activities, with previous studies reporting it to have potential value against breast cancer (Wang et al. 2012; Chen et al. 2015), prostate cancer (Pitchakarn et al. 2013; Vicinanza et al. 2013), bladder cancer (Ceci et al., 2016) and human osteogenic sarcoma (Han et al. 2006). In the present study, EA was found to be present at a concentration of 518.11 µg g^−1^ DW in the MtRV extract, which may reinforce the anti-tumor activity of BA.

Also, the rutin and chlorogenic acid present in MtRV extract, in the current study (257.90 µg g^−1^ and 177.34 dry weight, respectively), may exhibit anti-tumor properties similar to those described for other phenolic compounds (Chen et al. 2013; Yan et al. 2015; Zhang et al. 2015a, b; ben Sghaier et al. 2016; Gouthamchandra et al. 2017). Many studies indicate that phenolic compounds may play an important role in the prevention and treatment of cancer, and that natural, plant-derived compounds, do not typically demonstrate side effects, which is extremely important in cancer therapy.

## Conclusions

The present study is the first to demonstrate the anti-cancer properties of root extract of *M. trifoliata* plants derived from in vitro against grade IV glioma cells. Our findings demonstrate that polyphenolic compounds (e.g. ellagic acid, hydroxybenzoic acid, syringic acid, sinapinic acid, chlorogenic acid, ferulic acid or rutin) and terpenoids (betulinic acid) present in this extract can exert a synergistic effect and induce apoptosis by G2/M phase cell cycle arrest, change the protein expression level of Bax, Bcl-2, Cas-3 and p53 and decreased mitochondrial membrane potential in grade IV glioma cells. Our further research will focus on demonstrating the other properties of this plant extract and its potential application in practice.
